# Low microbiome diversity in threatened amphibians from two biodiversity hotspots

**DOI:** 10.1186/s42523-022-00220-w

**Published:** 2022-12-29

**Authors:** Sasha E. Greenspan, Pedro Peloso, Jesualdo A. Fuentes-González, Molly Bletz, Mariana L. Lyra, Ibere F. Machado, Renato A. Martins, Daniel Medina, Diego Moura-Campos, Wesley J. Neely, Jackson Preuss, Marcelo J. Sturaro, Renata I. Vaz, Carlos A. Navas, Luís Felipe Toledo, Alexandro M. Tozetti, Miguel Vences, Douglas C. Woodhams, Célio F. B. Haddad, Jason Pienaar, C. Guilherme Becker

**Affiliations:** 1 Department of Biological Sciences, The University of Alabama, Tuscaloosa, AL 35487 USA; 2 Programa de Pós Gradução em Zoologia, Universidade Federal do Pará/Museu Paraense Emílio Goeldi, Belém, Pará 66077-530 Brazil; 3 Instituto Boitatá de Etnobiologia e Conservação da Fauna, Goiânia, Goiás 74085-480 Brazil; 4 The Department of Biology and the Institute of Environment, Florida International University, Miami, FL 33199 USA; 5 Department of Biology, University of Massachusetts Boston, Boston, MA 02125 USA; 6 Department of Biodiversity and Aquaculture Center (CAUNESP), Universidade Estadual Paulista, Rio Claro, São Paulo 13506-900 Brazil; 7 Programa de Pós-Graduação em Conservação da Fauna, Universidade Federal de São Carlos, São Carlos, São Paulo 13565-905 Brazil; 8 Sistema Nacional de Investigación, SENACYT; City of Knowledge, Clayton, Panama, Republic of Panama; 9 Department of Biology, The Pennsylvania State University, University Park, PA 16803 USA; 10 Laboratório de História Natural de Anfíbios Brasileiros (LaHNAB), Departamento de Biologia Animal, Instituto de Biologia, Universidade Estadual de Campinas, Campinas, São Paulo 13083-862 Brazil; 11 Division of Ecology and Evolution, Research School of Biology, The Australian National University, Canberra, 2601 Australia; 12 Departamento de Ciências da Vida, Universidade do Oeste de Santa Catarina, São Miguel Do Oeste, Santa Catarina 89900-000 Brazil; 13 Departamento de Ecologia e Biologia Evolutiva, Universidade Federal de São Paulo, Diadema, São Paulo 09972-270 Brazil; 14 Departamento de Fisiologia Geral, Instituto de Biociencias, Universidade de São Paulo, São Paulo, São Paulo 05508-090 Brazil; 15 Programa de Pos-Graduacão em Biologia, Universidade do Vale do Rio dos Sinos, São Leopoldo, Rio Grande Do Sul 93022-750 Brazil; 16 Zoological Institute, Braunschweig University of Technology, Mendelssohnstr. 4, Brunswick, Germany

**Keywords:** Anuran, Endangered species, Host-associated microbial diversity, Brazil, Madagascar

## Abstract

Microbial diversity positively influences community resilience of the host microbiome. However, extinction risk factors such as habitat specialization, narrow environmental tolerances, and exposure to anthropogenic disturbance may homogenize host-associated microbial communities critical for stress responses including disease defense. In a dataset containing 43 threatened and 90 non-threatened amphibian species across two biodiversity hotspots (Brazil’s Atlantic Forest and Madagascar), we found that threatened host species carried lower skin bacterial diversity, after accounting for key environmental and host factors. The consistency of our findings across continents suggests the broad scale at which low bacteriome diversity may compromise pathogen defenses in species already burdened with the threat of extinction.

**Supplementary Information:**

The online version contains supplementary material available at 10.1186/s42523-022-00220-w.

## Main

Biodiversity loss is a major engine of ecosystem change in the Anthropocene [1]. The holobiont concept, in which the host and its microbiota function as a single organism, broadens the scales at which organisms and extinction drivers interact. The host microbiome is essential to immunity, development, metabolism, and stress responses, deeming it vitally interlinked with threatened species conservation. As a result, the targets of conservation biology have been expanding beyond preserving taxonomic and genetic diversity of species to include host-associated microbial diversity [2–4].

Species richness of host-associated bacterial assemblages (bacteriome) can exceed optimal levels, relative to host taxon and ecological context, if the microbiome is overwhelmed with transient or opportunistically pathogenic taxa [5]. However, in general, high bacterial diversity facilitates bacteriome stability, resilience, and function through mechanisms such as functional redundancy and synergy [6]. For instance, dramatic increases in metabolic, immune, and cognitive diseases since the twentieth century have been attributed to a consistent decline in human-associated microbiota diversity linked with industrialization [7].

Species more vulnerable to extinction may share certain biological traits, including habitat specialization, narrow environmental tolerances and geographic distributions, poor dispersal ability, and low genetic diversity [8]. These host traits, along with the habitat disturbances fueling species endangerment, may radically influence host bacteriomes, potentially skewing the bacteriome toward less diverse communities that may be less likely to support the symbiotic relationships and functions critical to host fitness and survival under the stresses of global change. However, the links between host endangerment and microbial diversity are unresolved across the host tree of life [9].

Amphibians stand apart from other animal groups as the most threatened taxon, with an estimated 41% of species listed in IUCN Red List threat categories, and are disproportionately impacted by emerging fungal, viral and protozoan diseases [10]. As in other taxa, amphibian bacteriome diversity often contributes to defenses against invading pathogens [11]. We tested the association between threat status and the taxonomic and phylogenetic diversity of skin-associated bacteria in 133 amphibian species (1454 samples) in two biodiversity hotspots nearly 10,000 km apart (Brazil’s Atlantic Forest and Madagascar). We compiled and analyzed data from 43 species listed under IUCN Red List threat categories (Brazil: 49 samples from nine species; Madagascar: 301 samples from 34 species) and 90 co-occurring non-threatened species (Brazil: 354 samples from 24 species; Madagascar: 750 samples from 66 species) across 63 localities (Brazil: 22 localities; Madagascar: 41 localities), defined as sampling areas less than 50 m in radius with consistent vegetation cover and microclimatic conditions. We targeted samples collected in natural forest habitat to control for direct effects of landcover. To standardize spatial variables, we averaged bacterial diversity metrics for each species within each sampling locality (Brazil: n = 56 species-localities; Madagascar: n = 278 species-localities; mean sample size per species-locality = 5.4 [Brazil/threatened], 7.5 [Brazil/non-threatened], 4.6 [Madagascar/threatened], 3.5 [Madagascar/non-threatened]). Threatened and non-threatened species spanned primary habitat use categories (aquatic, arboreal, terrestrial; see Methods) and were evenly distributed across the latitudinal sampling extent (Additional file 1: Fig. S1). We predicted decreasing skin bacteriome diversity with increasing threat status, based on risk factors for extinction such as specialization and narrow thermal breadth, and population characteristics of threatened species, including low genetic variation [12].

**Fig. 1:**
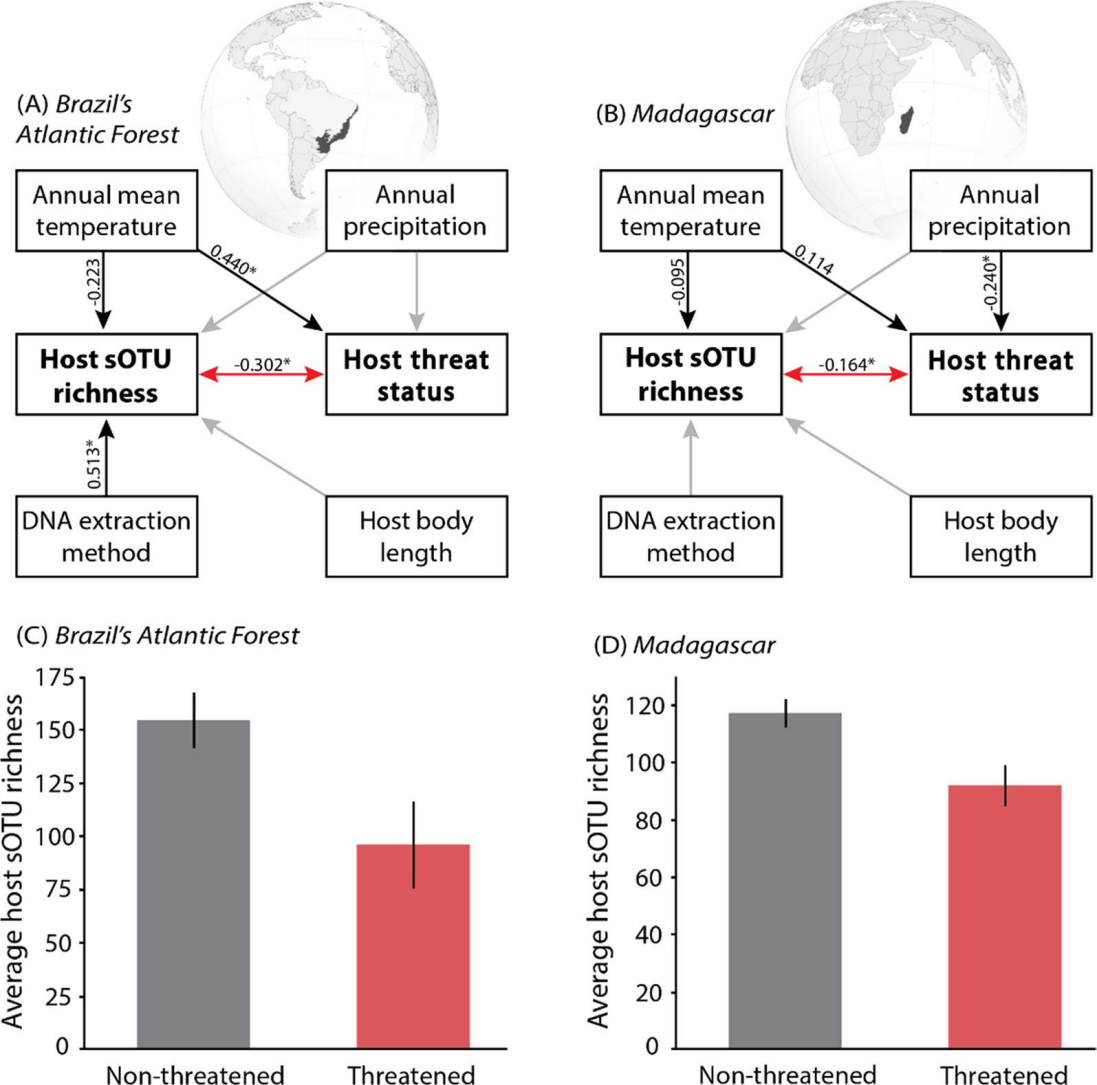
Association between skin bacterial diversity and threat status of amphibian species in two geographically distinct biodiversity hotspots. **A**, **B** Piecewise structural equation models accounting for environmental, host, and methodological factors. Amphibian skin bacterial diversity was estimated as number of detected sub-operational taxonomic units (sOTUs). Numbers are standardized coefficients (*p < 0.05). Unsupported paths shown as gray arrows were removed to improve model fit. Red arrows indicate the correlation of interest between host skin bacterial diversity and host threat status. Black arrows indicate other paths that were retained in the final pruned models. **C**, **D** Average skin sOTU richness between threatened (red) and non-threatened (gray) species. Threatened species carried lower skin sOTU richness in (**C**) Brazil’s Atlantic Forest (t = − 2.407, df = 15.436, *p* = 0.029) and **D** Madagascar (t = − 2.894, df = 131.981, *p* = 0.004). Error bars represent standard error

Our study reveals a cross-continental pattern of lower bacteriome diversity in the most threatened species within the most vulnerable animal taxon. Using a dual analysis approach including piecewise structural equation modeling and phylogenetic path analysis, we found that taxonomic diversity of host skin-associated bacteria (richness of sub-operational taxonomic units [sOTUs]) was negatively correlated with host threat status among amphibian communities in Brazil’s Atlantic Forest (Figs. 1A, C; Additional file 1: Table S1A) and Madagascar (Figs. 1B, D; Additional file 1: Table S1B). This correlation was robust to variation in climate, sample DNA extraction method (Fig. 1; Additional file 1: Table S1), host body length, vegetation density, and host geographic range area (Additional file 1: Table S2), and remained unaltered after accounting for host phylogeny (Additional file 1: Table S3) and host primary habitat type (Additional file 1: Fig. S2; see interaction term in Additional file 1: Table S4A). We found an identical pattern of lower Faith’s phylogenetic skin bacterial diversity in threatened compared to non-threatened species (Additional file 1: Fig. S3; Table S4B). In both focal geographic regions, threatened and non-threatened species carried differentially abundant skin bacterial taxa (Additional file 1: Figs. S4 and S5, Table S5), including higher abundances of the fungal pathogen-fighting skin bacterium *Janthinobacterium lividum* carried by non-threatened amphibian species in Brazil. Determining the drivers of these patterns will require disentangling host physiology, ecology and biogeography alongside the biotic and abiotic environmental stressors differentially affecting threatened and non-threatened species.

Ecological specialization may predispose species to endangerment. Many of the threatened species included in our study have relatively small distributions and specialized behaviors and physiologies. For example, the endangered treefrog *Xenohyla truncata*, endemic to coastal scrub forests in the Brazilian state of Rio de Janeiro, feeds on fruit (a unique trait among amphibians), in addition to small arthropods, and shelters in bromeliads [13]. Critically endangered toadlets *Melanophryniscus admirabilis* are confined to mossy rock pools along one river in southern Brazil [14]. The critically endangered Brazilian treefrog *Nyctimantis pomba* is found exclusively in bamboo groves within a single forest fragment [15]. In Madagascar, the microendemic ecological specialists *Gephyromantis corvus* and *G. kintana* are restricted to the Isalo sandstone massif, and the threatened montane specialists *Boophis laurenti*, *Mantidactylus pauliani*, *M. madecassus*, and *Anodonthyla montana* are each restricted to extremely narrow geographic ranges on the island's highest mountain peaks [16]. Together, these highly specialized host natural histories suggest high fidelity to specific microhabitats, dietary constraints, low dispersal capabilities and, in mild climates, narrow physiological tolerances that may limit the diversity of bacteria encountered in the environment and subsequently recruited to the skin microbiome.

Geographic range area was a poor predictor of skin bacterial diversity, potentially a reflection of our individual-level sampling scale. Compared to wide-ranging non-threatened species, threatened species with inherently small or shrinking geographic distributions may encounter smaller and potentially less diverse environmental source pools from which host microbiomes are seeded across the entire species range. However, our study controlled for direct spatial effects by sampling at a narrow, local scale (locality). Limiting our sampling to habitats with natural vegetation likewise accounted for direct effects of disturbance on host microbiomes and environmental bacterial source pools, such as microbiome depletion with increasing deforestation [17]. Indeed, threatened amphibian species are often only found in the most intact habitats [10] where environmental bacterial pools should be highly diverse [17], drawing an even stronger contrast between observed skin bacterial diversity of threatened species and expected diversity based on meso- or macro-scale habitat characteristics.

In the case of some endemic species with restricted geographic distributions, however, encroaching anthropogenic activity could impose physiological stress that may account for lower skin bacterial diversity, such as microclimatic edge effects or environmental contamination. For instance, the critically endangered *Physalaemus soaresi* is restricted to a single natural forest fragment less than 5 km^2^ in area within the suburbs of Rio de Janeiro [18]. Thus, factors associated with environmental degradation such as pollution, altered food web dynamics, and climatic shifts from nearby development could generate physiological stress through impacts on energetics, food quality and availability, behavior, and environmental microbial pools, with potential downstream shifts in skin microbiome composition [19]. Host population bottlenecks commonly experienced by threatened species in stress-generating environments may also constrain bacteriome diversity through sampling effects from generation to generation [20].

We found that threatened amphibian species from two continents carried lower skin bacterial diversity than non-threatened species. This pattern was consistent across all three primary host habitat use categories (arboreal, aquatic, terrestrial) and was robust to climate, sample DNA extraction method, host body length, vegetation density, host geographic range area, and host phylogeny. Regardless of the cause-and-effect associations between low bacteriome diversity and endangerment, this pattern has implications for health and fitness of threatened vertebrates. Diversity of the bacteriome may enhance resilience to invasion, environmental change, and other stressors through functional redundancy, partitioning use of limiting resources, or production of antimicrobial metabolites [21]. Another finding from the Brazil dataset that deserves further attention is that non-threatened amphibian species not only carried higher skin bacterial diversity than threatened species but also carried higher abundances of *Janthinobacterium lividum*. This amphibian skin bacterium has known inhibitory function against the causative agent of the amphibian fungal disease chytridiomycosis and has been successfully employed as a probiotic in amphibians susceptible to this disease [22]. Given that species are at increased risk of disease and other pressures as they move toward extinction [23], our findings suggest that the combined threats of low microbial diversity and vulnerability to pathogens may compound extinction risk, especially considering the slow pace of bacteriome recovery following pathogenic infections or other environmental disturbances [24, 25]. Our results raise a red flag for amphibians and threatened taxa from other animal groups and highlight the need for mechanistic insights to explain the geographically consistent and statistically robust inverse association between host threat status and bacteriome diversity.

There is some evidence that amphibian species driven to endangerment by disease can develop resistant microbiomes in response to pathogen pressure [26]. Alternatively, microbiome manipulation can mimic these processes interventionally [27]. However, whether naturally low bacteriome diversity hampers adaptive immune processes or efficacy of probiotic strategies is unresolved. Also, critical to threatened species conservation is the intersection of the host bacteriome and increasingly extreme temperature fluctuations associated with anthropogenic climate change, one of the most serious threats to listed species [28]. Our findings bring to light the urgency in characterizing microbial baselines for threatened species, not only as reference points for probiotics, but also to maximize success of wildlife ‘ark’ programs. Within these programs, bacteriome surveillance can guide captive husbandry protocols, antibiotic use, early detection of disease outbreaks and other environmental disturbances, translocation and reintroduction programs, and can be used as a metric for assessing efficacy of habitat restoration.

## Methods

### Study regions and sampling design

We conducted this study with data from two tropical biodiversity hotspots: Brazil’s Atlantic Forest and Madagascar (Additional file 2). The Brazil database comprised sequence data from nine previously published [29–33] and unpublished studies (SRA Bioproject accession PRJNA767814). This database consisted of samples from 403 individual post-metamorphic anurans representing 33 species (9 threatened; 24 non-threatened) and eight families from 22 localities (Additional file 1: Fig. S1). The Madagascar database comprised sequence data from one previously published study [16, 34] (SRA Bioproject accession PRJNA394790). This database consisted of samples from 1,051 individual post-metamorphic anurans representing 100 species (34 threatened; 66 non-threatened) and four families from 41 localities (Additional file 1: Fig. S1).

Both countries were sampled across a broad geographic area to capture a wide breadth of amphibian species diversity, with sampling localities haphazardly distributed (Additional file 1: Fig. S1). Threatened and non-threatened species were evenly distributed across the latitudinal sampling extent and frequently co-occurred within localities (Additional file 1: Fig. S1). We further ruled out spatial bias in our sampling by estimating potential differences in spatial autocorrelation of sOTU richness across distance bands for threatened versus non-threatened amphibian species using Moran’s I correlograms (Additional file 1: Fig. S6). Threatened and non-threatened species both spanned primary habitat use categories (species per category = 2 terrestrial, 3 arboreal, 4 aquatic [Brazil/threatened]; 8 terrestrial, 7 arboreal, 9 aquatic [Brazil/non-threatened]; 16 terrestrial, 9 arboreal, 9 aquatic [Madagascar/threatened]; 22 terrestrial, 32 arboreal, 12 aquatic [Madagascar/non-threatened]).

### Sampling protocol

Anurans were captured in the wild in localities with primarily natural vegetation, handled using disposable gloves, rinsed with sterile water to remove transient microbes and debris, and the skin surface was swabbed using sterile swabs following a standard protocol [35]. Swabs were kept on ice in the field and stored frozen at − 20 °C until further processing. Samples were primarily collected during the Austral breeding season from September to March, with a small proportion of samples collected in northern Brazil during the boreal breeding season in June.

### Bacterial sequencing and bioinformatics

Bacterial DNA was extracted from swabs from Brazil using the Qiagen DNeasy Blood and Tissue Kit or Prepman Ultra. DNA was extracted from swabs from Madagascar using the Qiagen DNeasy PowerSoil kit. DNA was amplified, purified, and sequenced on Illumina MiSeq sequencing platforms (2 × 250 or 2 × 150) following the Earth Microbiome Project 16S Illumina Amplicon Protocol. This protocol targets the V4 region of the 16S rRNA gene from the bacterial genome using barcoded primers 515F and 806R and a dual index approach.

The Brazil dataset was processed similarly to the previously published Madagascar dataset [16, 34]. Sequences were initially processed with Quantitative Insights into Microbial Ecology (Brazil: QIIME 2 version 2019.1 [36]; Madagascar: QIIME [34]). To maximize read quality [37], only forward reads trimmed to 150 base pairs were used for both datasets. Sequences were filtered based on quality score, using the q-score plugin for Brazil sequences and analogous criteria for Madagascar sequences [34]. Quality-filtered sequences were clustered into sub-operational taxonomic units (sOTUs) using the Deblur workflow [38]. Phylogenetic trees were constructed using FastTree and taxonomy was assigned with the classify‐sklearn naïve Bayes taxonomy classifier and the Greengenes 13.8 reference sequence database (Brazil) or the Ribosomal Database Project Classifier 5 with a custom script (Madagascar) [34]. Rare sOTUs were filtered by discarding sOTUs with less than 0.001% (53 reads) of total sequence reads across the dataset (Brazil) or less than 10 sequence reads across the dataset (Madagascar). We used conservative thresholds for removal of rare sOTUs because our datasets spanned large geographic areas and many host species, and thus a large proportion of sOTUS were relatively rare. For the Brazil samples, we discarded one sOTU that was considered a contaminant because it was abundant across control and template DNA samples. For the Madagascar samples, potential contaminants were identified with the group_significance.py script in QIIME and discarded [34].

After filtering and decontamination, the Brazil dataset contained 4,823,452 reads and an average of 11,969 reads per sample and the Madagascar dataset contained 18,084,933 reads and an average of 17,207 reads per sample. To normalize read counts across samples, samples were rarefied to 1500 (Brazil) or 2500 (Madagascar) and averaged 154 (Brazil) and 110 (Madagascar) sOTUs per sample [39]. We examined rarefaction curves to ensure sufficient sequence depth (Additional file 1: Figs. S7 and S8).

To ensure that our data were not biased by using only forward sequence reads, we repeated the Brazil bioinformatics using both forward and reverse sequence reads (QIIME 2 version 2021.8). Forward and reverse reads were joined for compatibility with the Deblur workflow [38]. We used the same filtering (0.001% of total sequence reads across dataset = 40) and decontamination methods as described for the Brazil dataset above. After filtering and decontamination, this dataset contained 3,632,608 reads and an average of 7762 reads per sample. To normalize read counts across samples, samples were rarefied to 500 and averaged 77 sOTUs per sample [39]. The lower rarefaction threshold (500) compared to the threshold used with only forward reads (1500) reflects consistently lower numbers of sequence reads detected per sample when using joined forward and reverse reads, likely due to lower quality of reverse reads. However, we examined rarefaction curves to ensure that this rarefaction level captured sufficient sequence depth (Additional file 1: Fig. S9). After rarefaction, the dataset contained 401 samples (49 threatened; 352 non-threatened), a similar sample size to the 403 samples (49 threatened; 354 non-threatened) analyzed when using only forward sequence reads.

### Statistical analysis

We analyzed the Brazil and Madagascar datasets separately. We used piecewise structural equation models (psem function in the piecewiseSEM package in Program R) to test for correlations between IUCN threat status and sOTU richness while accounting for direct and indirect associations with key environmental and host factors [40, 41]. We averaged sOTU richness for each species within each sampling locality (Brazil: n = 56 species-localities; Madagascar: n = 278 species-localities; mean sample size per species-locality = 5.4 [Brazil/threatened], 7.5 [Brazil/non-threatened], 4.6 [Madagascar/threatened], 3.5 [Madagascar/non-threatened]). We classified species into threat categories using a Brazil-specific Red List [42] and the International Union for Conservation of Nature’s (IUCN) Red List of Threatened Species for Madagascar species [10]. We classified threat status as a binary variable with Least Concern species assigned a value of 0 and threatened (Vulnerable, Endangered, and Critically Endangered) and Near-Threatened (NT) species assigned a value of 1 [10]. We grouped threatened and NT species because almost all NT species are reported to be impacted by land use change [10, 43, 44].

Environmental covariates included annual mean temperature (BIO1) and annual precipitation (BIO12), extracted for each sampling locality from WorldClim at 5-min spatial resolution, and Normalized Difference Vegetation Index (NDVI), averaged over the sampling period for each locality. Host species covariates included body length (maximum male snout-vent length [SVL])[45] and geographic range area (log-transformed). For Brazil, we included DNA extraction method as an additional predictor of sOTU richness to account for variation attributable to differences in DNA extraction protocol among samples. Preliminary generalized linear models (Poisson error distribution, log link) revealed that NDVI and geographic range area were consistently poor predictors of sOTU richness for both the Brazil and Madagascar datasets and were excluded from SEMs (Additional file 1: Table S2). Results of preliminary models remained unaltered as standard least squares general linear models, so we report the more conservative Poisson models. For the SEMs, we first ran saturated models with all ecologically relevant paths and then ran simplified models with unsupported variables excluded to improve model fit based on Akaike’s Information Criterion (AIC; Brazil: saturated model AIC = 21.591, simplified model AIC = 15.108; Madagascar: saturated model AIC = 33.550, simplified model AIC = 14.612). To ensure data were not biased by using only forward sequence reads, we repeated SEMs for Brazil using joined forward and reverse sequence data and our results remained unaltered (Additional file 1: Table S6).

To determine if SEM results were robust to effects of possible non-independence due to shared trends of common ancestry among frog species, we also tested the association between skin bacterial richness and threat status with phylogenetic path analysis (PPA) under the *d*-separation method [46] as implemented in the R package phylopath [47]. Each causal model was formulated in terms of separate directed acyclic graphs where conditional independencies (i.e. *d*-separation statements) were translated into phylogenetic linear regressions for analysis using phylolm [48]. This R package allows for measuring the degree of phylogenetic signal embedded in the data when the causal parents of the models are either continuous (*λ*) [49, 50] or discrete (*α*) [51]. In contrast to SEM, which accommodates a bidirectional link between skin bacterial richness and threat status, here we modeled averaged path coefficients shared by the alternative directed acyclic graphs, weighing them under the C-statistic information criterion corrected for small sample sizes (CIC_c_) which provides a measure of the strength of evidence for each whole path model [52].

The evolutionary correlations for PPA were accounted for by using the phylogenies from Hedges et al. [53] for the Brazil dataset, and Bletz et al. [16] for the Madagascar dataset. The former [53] corresponds to a time-calibrated amphibian tree synthesized from studies in molecular evolution and phylogenetics [54, 55], and pruned to include only our sampled Brazilian species. The latter [16] corresponds to the ultrametric time-tree computed with optimized branch lengths under the GTR model from 16S rRNA sequences. For the focal taxa, we compiled sequences of the 16S rRNA gene for all included taxa of Madagascar frogs, and aligned them with MAFFT v. 7 [56]. We then computed an initial phylogenetic tree under Maximum Likelihood and the general time reversible (GTR) substitution model in MEGA7 [57]. We then manually adjusted the topology of this tree to fit the most recent multi-gene phylogenetic trees available for subsets of these taxa, and for their deep relationships: Scherz et al. [58] for microhylids, Wollenberg et al. [59] for *Mantidactylus*, Kaffenberger et al. [60] for *Gephyromantis*, and Hutter et al. [61] for *Boophis*. We then optimized branch lengths of this user tree in MEGA7 under the GTR model. We also used the same program to compute an ultrametric time-tree from the same data set, using the RELTIME approach.

The negative correlation between threat status and skin bacterial diversity persisted after accounting for host phylogeny (Additional file 1: Table S3). The phylogenetic approach favored threat status as a predictor of bacterial diversity over the reverse. The phylogenetic analysis was consistent with the SEMs, except for one discrepancy for Madagascar. While SEM detected that abiotic effects on the correlation between threat status and skin bacterial diversity were primarily mediated through effects of temperature on threat status, PPA detected stronger effects of temperature on skin bacterial diversity.

We used general linear models as an additional tool to verify that known host factors did not influence the correlation between host threat status and skin bacterial diversity. For these models, we included host skin bacterial diversity (sOTU richness or Faith’s phylogenetic diversity) as the response and the following predictors: host primary habitat type (arboreal, aquatic, or terrestrial), threat status (threatened or non-threatened), country (Brazil or Madagasar), and the interaction between host primary habitat type and threat status.

We used linear discriminant analysis effect size (LEfSe) on the Galaxy platform to detect differentially abundant sOTUs between threatened and non-threatened species in each diversity hotspot [62, 63]. We used default parameters except increasing the threshold on the logarithmic LDA score (LogLDA) from 2.0 to 3.0 in order to remove weaker correlations. To visualize the results of this analysis we constructed heat maps using the heatmap.2 function in R (Heatplus package, R version 4.2.0) for Brazil and Madagascar data separately [64].

## Supplementary Information

**Additional file 1:** Supplemental figures and tables.

**Additional file 2:** List of samples and associated metadata.

## Data Availability

The datasets generated and analyzed during the current study are available in the SRA repository (Brazil: Bioproject accession PRJNA767814; Madagascar: Bioproject accession PRJNA394790).

## Abbreviations

AIC: Akaike’s information criterion
CIC_c_: C-statistic information criterion corrected for small sample sizes
GTR: General time reversible
IUCN: International union for conservation of Nature
LEfSe: Linear discriminant analysis effect size
NDVI: Normalized difference vegetation index
NT: Near-threatened
PPA: Phylogenetic path analysis
SEM: Structural equation model
sOTU: Sub-operational taxonomic unit
SVL: Snout-vent length
