# Supplementary material for: Low microbiome diversity in threatened amphibians from two biodiversity hotspots
Source: Anim Microbiome. 2022 Dec 29;4:69. doi: 10.1186/s42523-022-00220-w (PMC9801548; doi:10.1186/s42523-022-00220-w)
Supplement: Supplementary file 1 — Additional file 1: Supplemental figures and tables. [file 42523_2022_220_MOESM1_ESM.docx]

Supplementary Materials for

Low microbiome diversity in threatened amphibians from two biodiversity hotspots

**Authors:** Sasha E. Greenspan^*^, Pedro Peloso, Jesualdo A. Fuentes-González, Molly Bletz, Mariana L. Lyra, Ibere F. Machado, Renato A. Martins, Daniel Medina, Diego Moura-Campos, Wesley J. Neely, Jackson Preuss, Marcelo J. Sturaro, Renata I. Vaz, Carlos A. Navas, Luís Felipe Toledo, Alexandro M. Tozetti, Miguel Vences, Douglas C. Woodhams, Célio F. B. Haddad, Jason Pienaar, C. Guilherme Becker^*^

Correspondence to: [sasha.greenspan@gmail.com](mailto:sasha.greenspan@gmail.com), [guibecker@psu.edu](mailto:guibecker@psu.edu)


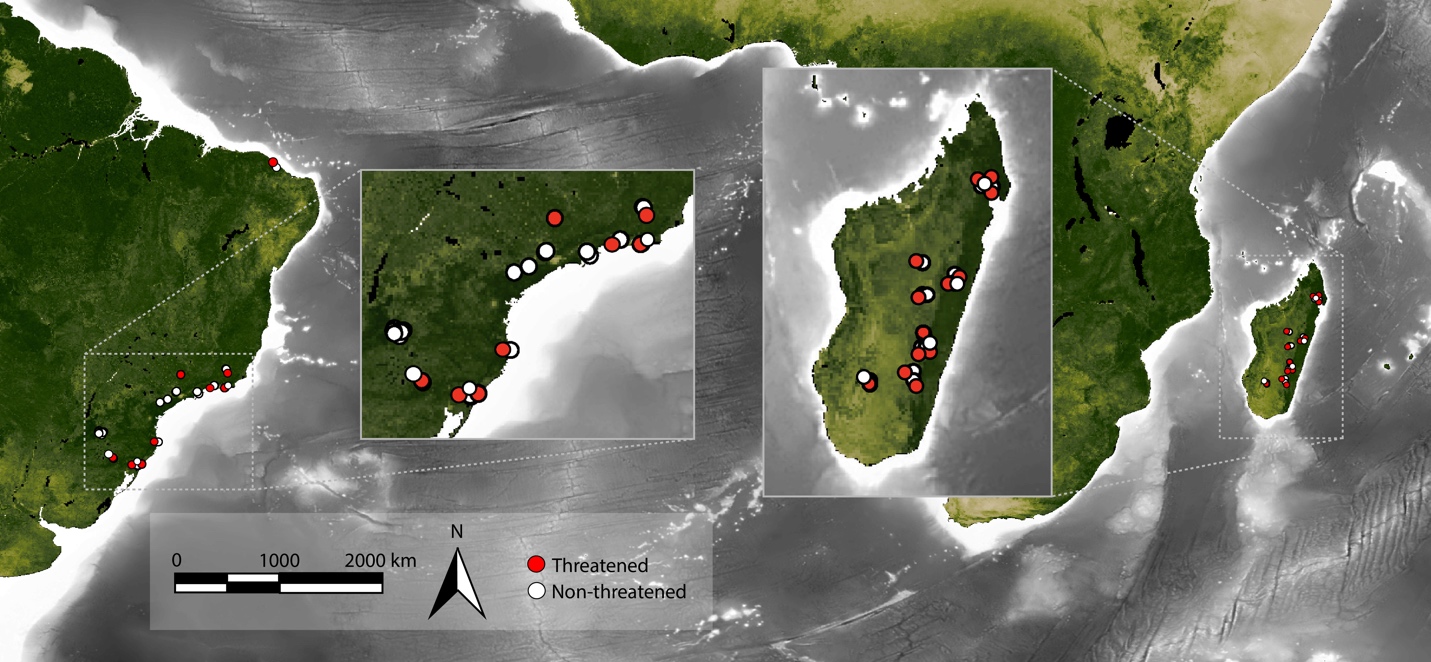


Fig. S1.

Sampling localities in Brazil’s Atlantic Forest and Madagascar.


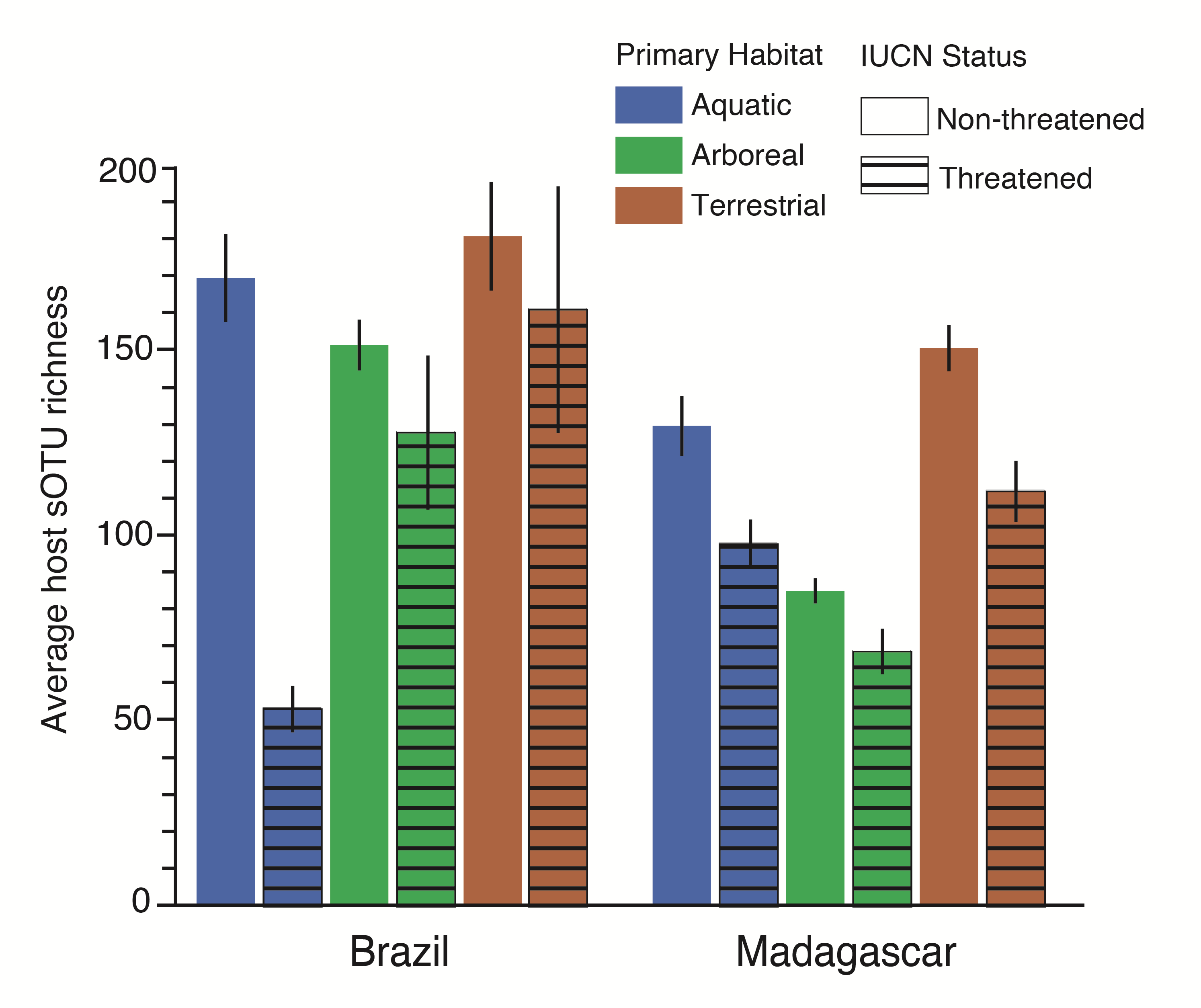


**Fig. S2.**

Average sOTU richness of the host bacteriome in threatened (bars with horizontal stripes) and non-threatened (bars without stripes) amphibians spanning three primary host habitat use categories (aquatic, arboreal, terrestrial). Error bars represent standard errors.


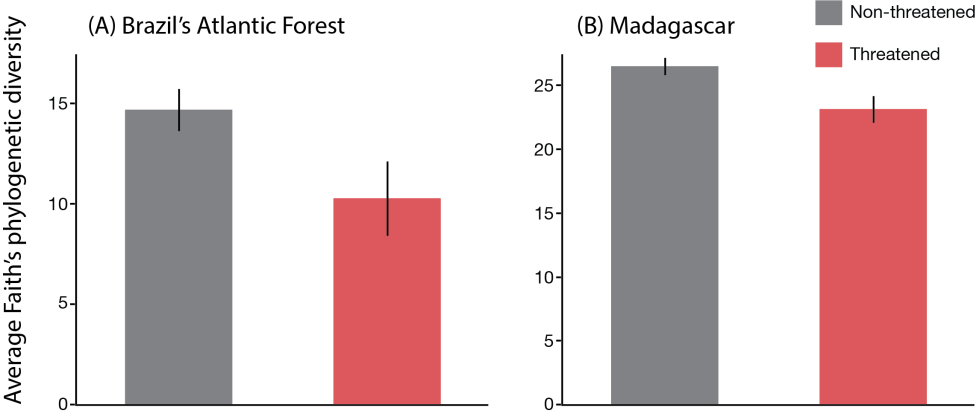


Fig. S3.

Average of Faith’s phylogenetic diversity of amphibian skin bacteria between threatened (red) and non-threatened (gray) species in two biodiversity hotspots. Threatened species carried lower phylogenetic diversity of skin bacteria in **(A)** Brazil’s Atlantic Forest (t = -2.074, df = 13.688, *p* = 0.057) and **(B)** Madagascar (t = -2.711, df = 122.649, *p* = 0.008). Error bars represent standard error.


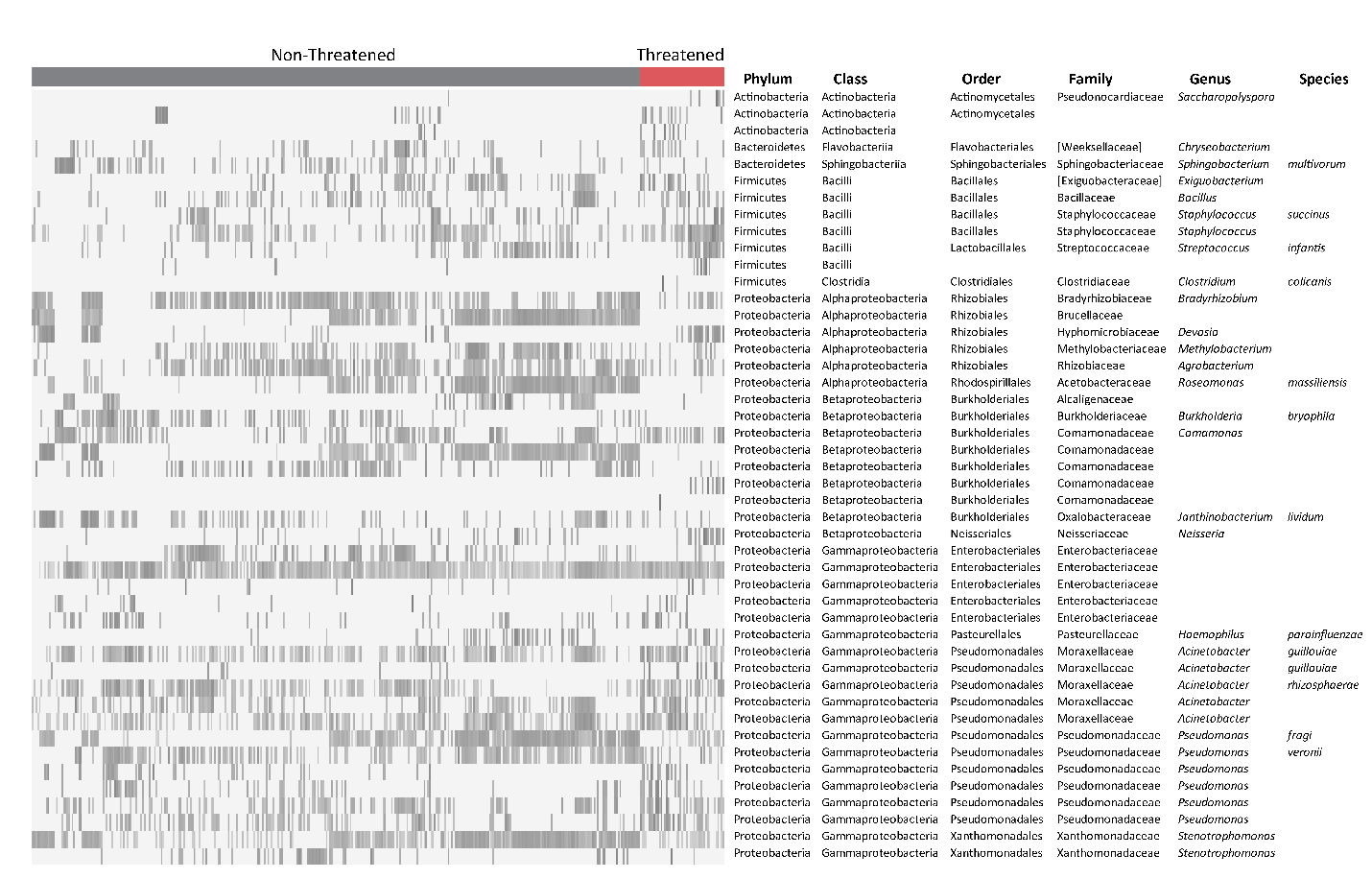


Fig. S4.

Heatmap showing differentially abundant bacterial taxa between threatened (red) and non-threatened (gray) amphibian species in Brazil’s Atlantic Forest. In total, 46 taxa were differentially abundant with 29 higher in threatened and 17 higher in non-threatened species. Darker shades of gray indicate greater relative abundance of the given taxa in that sample.


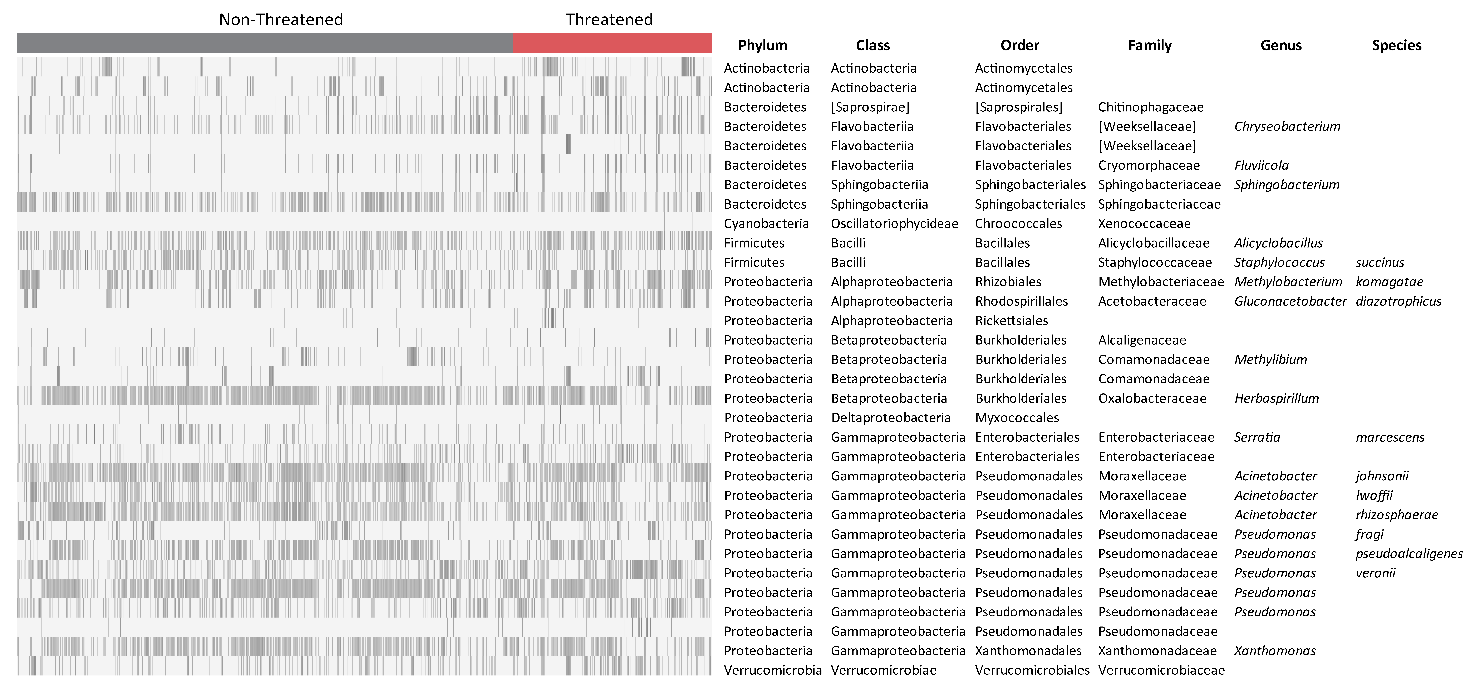


Fig. S5.

Heatmap showing differentially abundant bacterial taxa between threatened (red) and non-threatened (gray) amphibian species in Madagascar. In total, 32 taxa were differentially abundant with 23 higher in threatened and 9 higher in non-threatened species. Darker shades of gray indicate greater relative abundance of the given taxa in that sample.


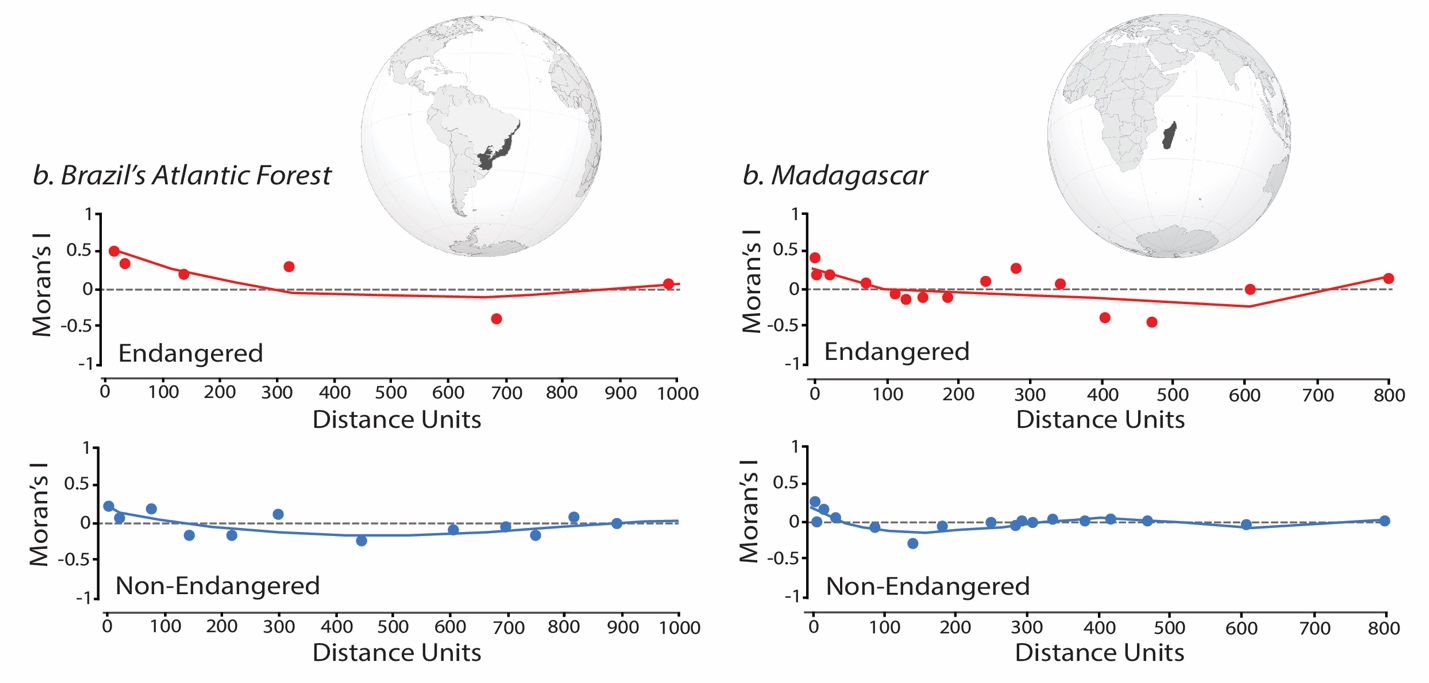


Non-threatened

Threatened

Threatened

Non-threatened

**Figure S6.**

Moran’s I correlograms indicating similar patterns of low spatial autocorrelation of sOTU richness between threatened and non-threatened amphibian species in Brazil’s Atlantic Forest (a) and Madagascar (b). Positive Moran’s I indicates similar sOTU richness values clustering together in space across distance units. Negative Moran’s I indicates contrasting sOTU richness values clustering together in space across distance units. Values approaching zero (indicated by the dashed line) denote lack of spatial autocorrelation. Solid line indicates average Moran’s I across distance units.


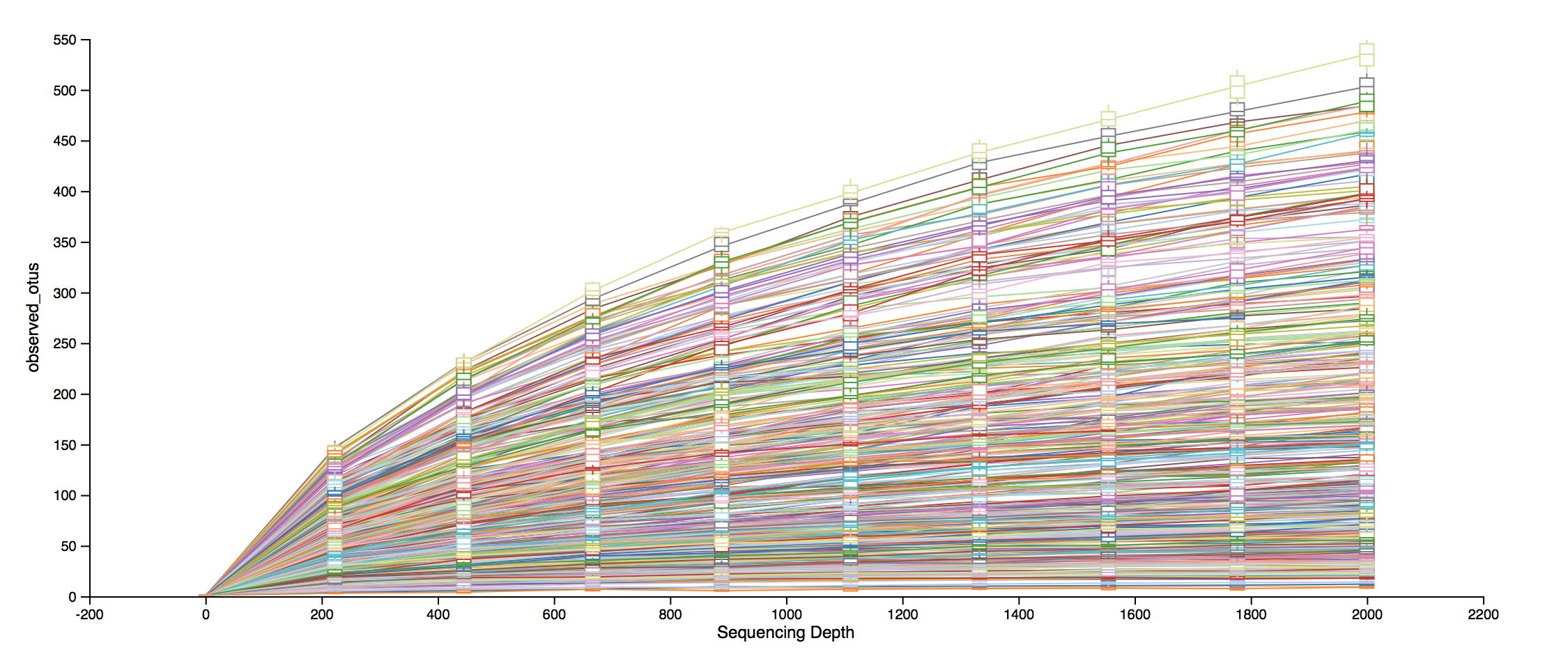


**Fig. S7.**

Rarefaction curves for Brazil data using forward sequence reads.


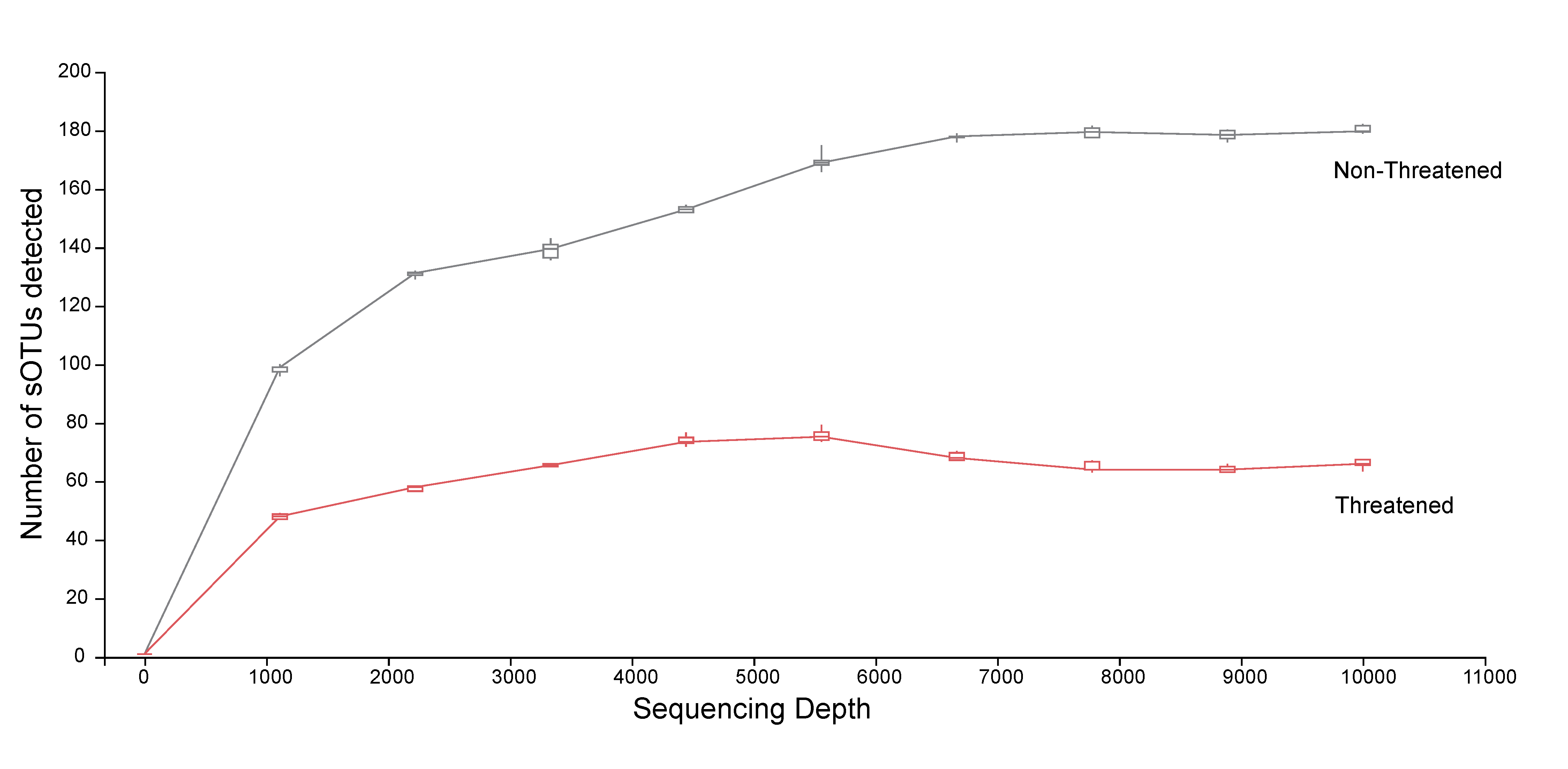


**Fig. S8.**

Grouped rarefaction curves for samples of threatened and non-threatened species from Brazil. At our selected sequencing depth of 1500, samples from threatened species have reached saturation, suggesting that lower OTU richness in threatened compared to non-threatened species is not an artifact of rarefaction.


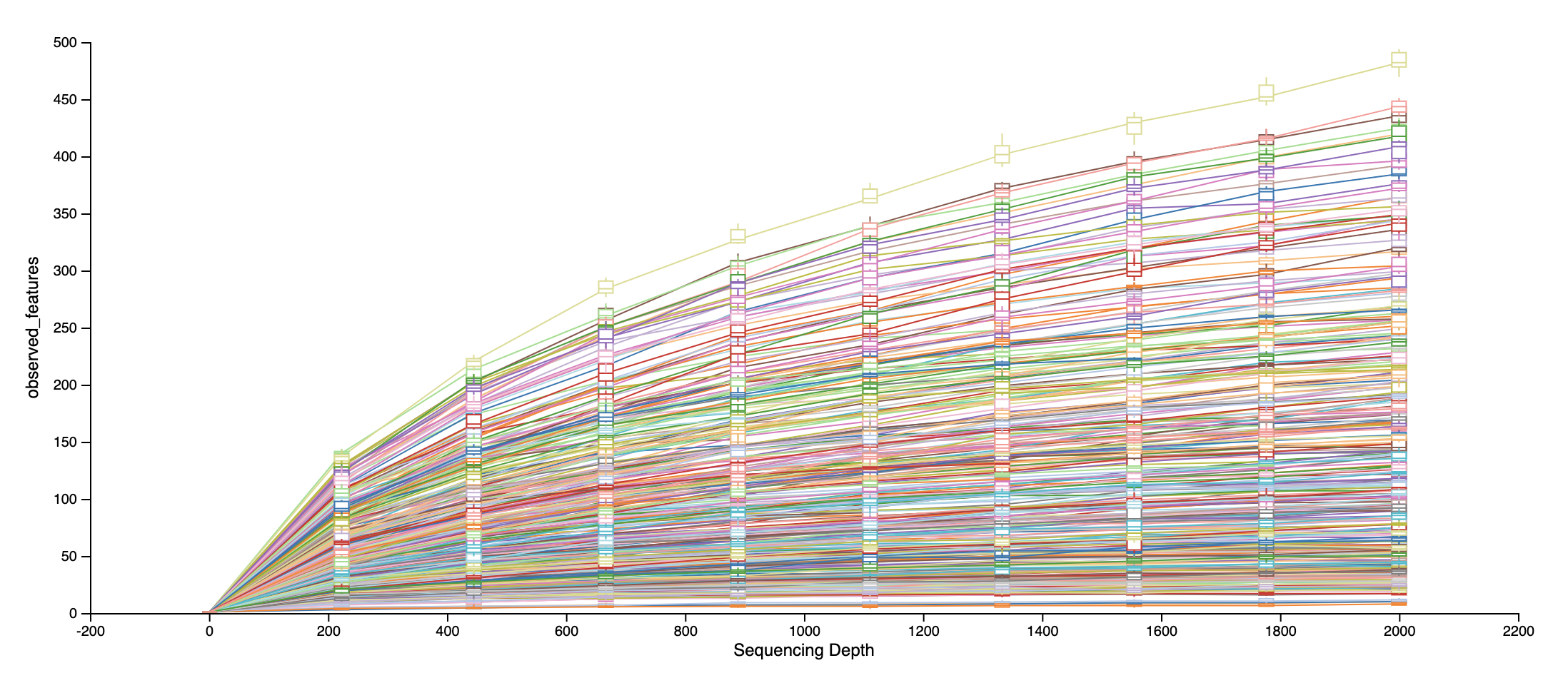


**Fig. S9.**

Rarefaction curves for Brazil data using joined forward and reverse sequence reads.

Table S1.

Results of simplified piecewise structural equation models (SEM). SEMs accommodated a bidirectional link between skin bacterial richness and threat status.

**(A) Brazil**

| Response | Predictor | Estimate | Std. error | | df | | Crit. Value | | *p* | | Std. estimate | |  |
| --- | --- | --- | --- | --- | --- | --- | --- | --- | --- | --- | --- | --- | --- |
| **Threat status** | **Annual mean temperature** | **0.007** | | **0.002** | | **54** | | **3.597** | | **0.001** | | **0.440** | |
| sOTU richness | Annual mean temperature | -0.823 | | 0.447 | | 53 | | -1.843 | | 0.071 | | -0.223 | |
| **sOTU richness** | **DNA extraction method** | **135.547** | | **31.945** | | **53** | | **4.243** | | **0.000** | | **0.513** | |
| **sOTU richness** | **Threat status (correlation)** | **-0.302** | |  | | **56** | | **-2.307** | | **0.013** | | **-0.302** | |

Whole model: Fisher's C = 1.108; *p* = 0.575; AIC = 15.108

**(B) Madagascar**

| Response | Predictor | Estimate | Std. error | | df | | Crit. Value | | *p* | | Std. estimate | |  |
| --- | --- | --- | --- | --- | --- | --- | --- | --- | --- | --- | --- | --- | --- |
| Threat status | Annual mean temperature | 0.020 | | 0.015 | | 275 | | 1.353 | | 0.177 | | 0.114 | |
| **Threat status** | **Annual precipitation** | **-0.001** | | **0.001** | | **275** | | **-2.849** | | **0.005** | | **-0.240** | |
| sOTU richness | Annual mean temperature | -2.797 | | 1.768 | | 276 | | -1.583 | | 0.115 | | -0.095 | |
| **sOTU richness** | **Threat status (correlation)** | **-0.164** | |  | | **278** | | **-2.764** | | **0.003** | | **-0.164** | |

Whole model: Fisher's C = 0.612; *p* = 0.736; AIC = 14.612

Table S2.

Results of preliminary generalized linear models (Poisson error distribution, log link). NDVI and geographic range area were consistently poor predictors of sOTU richness and were excluded from subsequent SEMs.

**(A) Brazil (response = sOTU richness)**

| Predictor | Estimate | Std. error | χ^2^ | *p* |
| --- | --- | --- | --- | --- |
| **Intercept** | **6.619** | **1.583** | **17.065** | **<0.0001** |
| Threat status | 0.256 | 0.157 | 2.757 | 0.097 |
| Annual mean temperature | -0.007 | 0.004 | 2.442 | 0.118 |
| Annual mean precipitation | -0.001 | 0.001 | 2.871 | 0.090 |
| NDVI | -0.219 | 0.854 | 0.065 | 0.798 |
| Average body length (SVL) | -0.003 | 0.002 | 1.786 | 0.181 |
| Geographic range area | 0.011 | 0.064 | 0.032 | 0.858 |
| **DNA extraction method** | **0.768** | **0.198** | **20.874** | **<0.0001** |

Whole model: χ^2^ = 39.498; df = 7; *p* = <0.0001

**(B) Madagascar (response = sOTU richness)**

| Predictor | Estimate | Std. error | χ^2^ | *p* |
| --- | --- | --- | --- | --- |
| **Intercept** | **4.936** | **0.435** | **98.746** | **<0.001** |
| **Threat status** | **0.114** | **0.058** | **3.964** | **0.047** |
| Annual mean temperature | -0.016 | 0.023 | 0.513 | 0.474 |
| Annual mean precipitation | -0.001 | 0.001 | 0.675 | 0.411 |
| NDVI | 0.029 | 0.361 | 0.007 | 0.935 |
| Average body length (SVL) | 0.001 | 0.002 | 0.125 | 0.724 |
| Geographic range area | 0.025 | 0.055 | 0.207 | 0.649 |

Whole model: χ^2^ =11.238 ; df = 6; *p* = 0.081

Table S3.

Results of phylogenetic path analyses.

**(A) Brazil**

| Response | Predictor | Estimate | Std. error | | Lower CI | | Upper CI | |  |
| --- | --- | --- | --- | --- | --- | --- | --- | --- | --- |
| **Threat status** | **Annual mean temperature** | **1.044** | | **0.417** | | **0.228** | | **1.861** | |
| sOTU richness | Annual mean temperature | -0.033 | | 0.193 | | -0.412 | | 0.346 | |
| **sOTU richness** | **DNA extraction method** | **1.411** | | **0.399** | | **0.628** | | **2.193** | |
| **sOTU richness** | **Threat status** | **-0.903** | | **0.444** | | **-1.773** | | **-0.034** | |
| Threat status | sOTU richness | -0.237 | | 0.306 | | -0.836 | | 0.361 | |

**(B) Madagascar**

| Response | Predictor | Estimate | Std. error | | Lower CI | | Upper CI | |  |
| --- | --- | --- | --- | --- | --- | --- | --- | --- | --- |
| Threat status | Annual mean temperature | -0.022 | | 0.051 | | -0.121 | | 0.076 | |
| Threat status | Annual precipitation | 0.017 | | 0.040 | | -0.061 | | 0.096 | |
| **sOTU richness** | **Annual mean temperature** | **-0.159** | | **0.062** | | **-0.281** | | **-0.037** | |
| **sOTU richness** | **Threat status** | **-0.423** | | **0.150** | | **-0.717** | | **-0.128** | |
| Threat status | sOTU richness | -0.005 | | 0.017 | | -0.038 | | 0.028 | |

Table S4.

General linear models (GLM) testing for individual and interacting effects of host threat status and primary habitat type on host skin bacterial diversity . Country was included as a fixed effect. The results remained unaltered when country was included as a random effect (not shown).

**(A)**

| **Average sOTU richness** | | | |
| --- | --- | --- | --- |
| *Whole Model: F_(6,1447)_ = 31.63, R2 = 0.12, p < 0.0001* | | | |
| Term | SoS | F | *p* |
| Country | 522999.17 | 65.33 | **<0.0001** |
| Habitat | 395875.24 | 24.73 | **<0.0001** |
| Threat status | 316089.70 | 39.49 | **<0.0001** |
| Habitat x Threat status | 30540.16 | 1.91 | 0.1488 |

**(B)**

| **Average Faith's phylogenetic diversity** | | | |
| --- | --- | --- | --- |
| *Whole Model: F_(6,1447)_ = 66.56, R2 = 0.22, p < 0.0001* | | | |
| Term | SoS | F | *p* |
| Country | 31558.36 | 275.47 | **<0.0001** |
| Habitat | 5682.92 | 24.80 | **<0.0001** |
| Threat status | 4083.21 | 35.64 | **<0.0001** |
| Habitat x Threat status | 130.76 | 0.57 | 0.5653 |

Table S5.

Differentially abundant taxa detected through linear discriminant analysis effect size (LEfSe) for Brazil (A) and Madagascar (B). LogLDA score and p-value show strength of the relationship.

**(A) Brazil**

| Phylum | Class | Order | Family | Genus | Species | Group | LogLDA | *p* |
| --- | --- | --- | --- | --- | --- | --- | --- | --- |
| Proteobacteria | Alphaproteobacteria | Rhizobiales | Methylobacteriaceae | *Methylobacterium* |  | Non-threatened | 3.01 | 0.0131 |
| Proteobacteria | Alphaproteobacteria | Rhizobiales | Bradyrhizobiaceae | *Bradyrhizobium* |  | Non-threatened | 3.08 | 0.0003 |
| Proteobacteria | Gammaproteobacteria | Pseudomonadales | Pseudomonadaceae | *Pseudomonas* | *veronii* | Non-threatened | 3.18 | 0.0214 |
| Proteobacteria | Betaproteobacteria | Burkholderiales | Comamonadaceae |  |  | Non-threatened | 4.13 | >0.0001 |
| Proteobacteria | Alphaproteobacteria | Rhizobiales | Brucellaceae |  |  | Non-threatened | 3.62 | >0.0001 |
| Proteobacteria | Gammaproteobacteria | Pseudomonadales | Pseudomonadaceae | *Pseudomonas* | *fragi* | Non-threatened | 4.51 | 0.0010 |
| Bacteroidetes | Sphingobacteriia | Sphingobacteriales | Sphingobacteriaceae | *Sphingobacterium* | *multivorum* | Non-threatened | 3.12 | 0.0177 |
| Proteobacteria | Gammaproteobacteria | Xanthomonadales | Xanthomonadaceae | *Stenotrophomonas* |  | Non-threatened | 4.26 | >0.0001 |
| Proteobacteria | Gammaproteobacteria | Enterobacteriales | Enterobacteriaceae |  |  | Non-threatened | 3.59 | 0.0044 |
| Proteobacteria | Gammaproteobacteria | Enterobacteriales | Enterobacteriaceae |  |  | Non-threatened | 3.85 | 0.0150 |
| Proteobacteria | Alphaproteobacteria | Rhizobiales | Rhizobiaceae | *Agrobacterium* |  | Non-threatened | 3.18 | 0.0039 |
| Proteobacteria | Betaproteobacteria | Burkholderiales | Oxalobacteraceae | *Janthinobacterium* | *lividum* | Non-threatened | 3.18 | 0.0255 |
| Bacteroidetes | Flavobacteriia | Flavobacteriales | [Weeksellaceae] | *Chryseobacterium* |  | Non-threatened | 3.04 | 0.0100 |
| Proteobacteria | Betaproteobacteria | Burkholderiales | Comamonadaceae |  |  | Non-threatened | 3.01 | 0.0008 |
| Proteobacteria | Betaproteobacteria | Burkholderiales | Burkholderiaceae | *Burkholderia* | *bryophila* | Non-threatened | 3.04 | >0.0001 |
| Proteobacteria | Alphaproteobacteria | Rhodospirillales | Acetobacteraceae | *Roseomonas* | *massiliensis* | Non-threatened | 3.28 | >0.0001 |
| Proteobacteria | Betaproteobacteria | Burkholderiales | Alcaligenaceae |  |  | Non-threatened | 3.67 | 0.0091 |
| Firmicutes | Bacilli | Bacillales | Staphylococcaceae | *Staphylococcus* | *succinus* | Threatened | 3.01 | 0.0325 |
| Proteobacteria | Alphaproteobacteria | Rhizobiales | Hyphomicrobiaceae | *Devosia* |  | Threatened | 3.54 | >0.0001 |
| Proteobacteria | Gammaproteobacteria | Pseudomonadales | Moraxellaceae | *Acinetobacter* | *guillouiae* | Threatened | 4.33 | 0.0202 |
| Proteobacteria | Gammaproteobacteria | Enterobacteriales | Enterobacteriaceae |  |  | Threatened | 3.60 | 0.0079 |
| Proteobacteria | Betaproteobacteria | Burkholderiales | Comamonadaceae | *Comamonas* |  | Threatened | 3.19 | 0.0013 |
| Firmicutes | Bacilli | Bacillales | Bacillaceae | *Bacillus* |  | Threatened | 3.34 | 0.0153 |
| Proteobacteria | Gammaproteobacteria | Pseudomonadales | Moraxellaceae | *Acinetobacter* |  | Threatened | 3.90 | 0.0428 |
| Firmicutes | Bacilli | Lactobacillales | Streptococcaceae | *Streptococcus* | *infantis* | Threatened | 3.45 | 0.0005 |
| Proteobacteria | Gammaproteobacteria | Pseudomonadales | Moraxellaceae | *Acinetobacter* | *guillouiae* | Threatened | 3.04 | >0.0001 |
| Proteobacteria | Gammaproteobacteria | Pseudomonadales | Pseudomonadaceae | *Pseudomonas* |  | Threatened | 3.40 | 0.0004 |
| Firmicutes | Bacilli | Bacillales | Staphylococcaceae | *Staphylococcus* |  | Threatened | 4.29 | >0.0001 |
| Firmicutes | Bacilli | Bacillales | [Exiguobacteraceae] | *Exiguobacterium* |  | Threatened | 3.72 | 0.0034 |
| Actinobacteria | Actinobacteria | Actinomycetales |  |  |  | Threatened | 3.10 | >0.0001 |
| Proteobacteria | Betaproteobacteria | Neisseriales | Neisseriaceae | *Neisseria* |  | Threatened | 3.35 | >0.0001 |
| Proteobacteria | Gammaproteobacteria | Enterobacteriales | Enterobacteriaceae |  |  | Threatened | 3.49 | 0.0175 |
| Proteobacteria | Betaproteobacteria | Burkholderiales | Comamonadaceae |  |  | Threatened | 3.80 | >0.0001 |
| Firmicutes | Clostridia | Clostridiales | Clostridiaceae | *Clostridium* | *colicanis* | Threatened | 3.89 | 0.0001 |
| Proteobacteria | Gammaproteobacteria | Pseudomonadales | Pseudomonadaceae | *Pseudomonas* |  | Threatened | 3.67 | 0.0009 |
| Actinobacteria | Actinobacteria | Actinomycetales | Pseudonocardiaceae | *Saccharopolyspora* |  | Threatened | 3.69 | >0.0001 |
| Proteobacteria | Gammaproteobacteria | Pseudomonadales | Moraxellaceae | *Acinetobacter* | *rhizosphaerae* | Threatened | 3.87 | 0.0039 |
| Firmicutes | Bacilli |  |  |  |  | Threatened | 3.11 | >0.0001 |
| Proteobacteria | Betaproteobacteria | Burkholderiales | Comamonadaceae |  |  | Threatened | 3.25 | 0.0072 |
| Proteobacteria | Gammaproteobacteria | Pseudomonadales | Pseudomonadaceae | *Pseudomonas* |  | Threatened | 4.18 | 0.0008 |
| Proteobacteria | Gammaproteobacteria | Enterobacteriales | Enterobacteriaceae |  |  | Threatened | 3.54 | 0.0235 |
| Proteobacteria | Gammaproteobacteria | Pseudomonadales | Moraxellaceae | *Acinetobacter* |  | Threatened | 4.50 | 0.0065 |
| Proteobacteria | Gammaproteobacteria | Pasteurellales | Pasteurellaceae | *Haemophilus* | *parainfluenzae* | Threatened | 3.24 | 0.0409 |
| Actinobacteria | Actinobacteria |  |  |  |  | Threatened | 3.83 | >0.0001 |
| Proteobacteria | Gammaproteobacteria | Pseudomonadales | Pseudomonadaceae | *Pseudomonas* |  | Threatened | 4.28 | >0.0001 |
| Proteobacteria | Gammaproteobacteria | Xanthomonadales | Xanthomonadaceae | *Stenotrophomonas* |  | Threatened | 3.15 | 0.0474 |

**(B) Madagascar**

| Phylum | Class | Order | Family | Genus | Species | Group | LogLDA | *p* |
| --- | --- | --- | --- | --- | --- | --- | --- | --- |
| Bacteroidetes | Sphingobacteriia | Sphingobacteriales | Sphingobacteriaceae |  |  | Non-threatened | 3.78 | >0.0001 |
| Proteobacteria | Gammaproteobacteria | Pseudomonadales | Pseudomonadaceae | *Pseudomonas* | *pseudoalcaligenes* | Non-threatened | 3.07 | >0.0001 |
| Proteobacteria | Gammaproteobacteria | Pseudomonadales | Moraxellaceae | *Acinetobacter* | *johnsonii* | Non-threatened | 3.27 | 0.0175 |
| Proteobacteria | Gammaproteobacteria | Xanthomonadales | Xanthomonadaceae | *Xanthomonas* |  | Non-threatened | 3.09 | 0.0030 |
| Proteobacteria | Gammaproteobacteria | Pseudomonadales | Moraxellaceae | *Acinetobacter* | *rhizosphaerae* | Non-threatened | 3.70 | >0.0001 |
| Firmicutes | Bacilli | Bacillales | Staphylococcaceae | *Staphylococcus* | *succinus* | Non-threatened | 3.01 | 0.0026 |
| Proteobacteria | Gammaproteobacteria | Enterobacteriales | Enterobacteriaceae | *Serratia* | *marcescens* | Non-threatened | 3.02 | 0.0099 |
| Proteobacteria | Gammaproteobacteria | Pseudomonadales | Pseudomonadaceae | *Pseudomonas* |  | Non-threatened | 4.21 | >0.0001 |
| Proteobacteria | Betaproteobacteria | Burkholderiales | Comamonadaceae | *Methylibium* |  | Non-threatened | 3.52 | 0.0004 |
| Firmicutes | Bacilli | Bacillales | Alicyclobacillaceae | *Alicyclobacillus* |  | Threatened | 3.26 | 0.0101 |
| Bacteroidetes | Sphingobacteriia | Sphingobacteriales | Sphingobacteriaceae | *Sphingobacterium* |  | Threatened | 3.06 | >0.0001 |
| Proteobacteria | Gammaproteobacteria | Pseudomonadales | Pseudomonadaceae | *Pseudomonas* | *veronii* | Threatened | 4.42 | >0.0001 |
| Cyanobacteria | Oscillatoriophycideae | Chroococcales | Xenococcaceae |  |  | Threatened | 3.05 | 0.0255 |
| Proteobacteria | Alphaproteobacteria | Rhizobiales | Methylobacteriaceae | *Methylobacterium* | *komagatae* | Threatened | 3.02 | 0.0001 |
| Bacteroidetes | Flavobacteriia | Flavobacteriales | [Weeksellaceae] |  |  | Threatened | 3.06 | >0.0001 |
| Verrucomicrobia | Verrucomicrobiae | Verrucomicrobiales | Verrucomicrobiaceae |  |  | Threatened | 3.30 | >0.0001 |
| Proteobacteria | Alphaproteobacteria | Rickettsiales |  |  |  | Threatened | 3.69 | >0.0001 |
| Proteobacteria | Deltaproteobacteria | Myxococcales |  |  |  | Threatened | 3.09 | 0.0301 |
| Proteobacteria | Gammaproteobacteria | Enterobacteriales | Enterobacteriaceae |  |  | Threatened | 3.63 | 0.0058 |
| Proteobacteria | Alphaproteobacteria | Rhodospirillales | Acetobacteraceae | *Gluconacetobacter* | *diazotrophicus* | Threatened | 3.11 | >0.0001 |
| Proteobacteria | Gammaproteobacteria | Pseudomonadales | Moraxellaceae | *Acinetobacter* | *lwoffii* | Threatened | 3.27 | 0.0025 |
| Proteobacteria | Betaproteobacteria | Burkholderiales | Oxalobacteraceae | *Herbaspirillum* |  | Threatened | 3.09 | >0.0001 |
| Actinobacteria | Actinobacteria | Actinomycetales |  |  |  | Threatened | 4.12 | >0.0001 |
| Proteobacteria | Betaproteobacteria | Burkholderiales | Alcaligenaceae |  |  | Threatened | 3.55 | 0.0030 |
| Proteobacteria | Gammaproteobacteria | Pseudomonadales | Pseudomonadaceae | *Pseudomonas* | *fragi* | Threatened | 3.34 | 0.0357 |
| Proteobacteria | Gammaproteobacteria | Pseudomonadales | Pseudomonadaceae | *Pseudomonas* |  | Threatened | 4.06 | 0.0001 |
| Actinobacteria | Actinobacteria | Actinomycetales |  |  |  | Threatened | 3.15 | 0.0066 |
| Proteobacteria | Betaproteobacteria | Burkholderiales | Comamonadaceae |  |  | Threatened | 3.32 | >0.0001 |
| Bacteroidetes | Flavobacteriia | Flavobacteriales | [Weeksellaceae] | *Chryseobacterium* |  | Threatened | 3.32 | 0.0019 |
| Bacteroidetes | Flavobacteriia | Flavobacteriales | Cryomorphaceae | *Fluviicola* |  | Threatened | 3.01 | >0.0001 |
| Bacteroidetes | [Saprospirae] | [Saprospirales] | Chitinophagaceae |  |  | Threatened | 3.62 | 0.0003 |
| Proteobacteria | Gammaproteobacteria | Pseudomonadales | Pseudomonadaceae |  |  | Threatened | 3.37 | >0.0001 |

Table S6.

Results of simplified piecewise structural equation model (SEM) for Brazil using forward and reverse sequence data. SEMs accommodated a bidirectional link between skin bacterial richness and threat status.

| Response | Predictor | Estimate | Std. error | | df | | Crit. Value | | *p* | | Std. estimate | |  |
| --- | --- | --- | --- | --- | --- | --- | --- | --- | --- | --- | --- | --- | --- |
| **Threat status** | **Annual mean temperature** | **0.007** | | **0.002** | | **54** | | **3.597** | | **0.001** | | **0.440** | |
| sOTU richness | Annual mean temperature | -0.379 | | 0.226 | | 53 | | -1.675 | | 0.100 | | -0.206 | |
| **sOTU richness** | **DNA extraction method** | **64.807** | | **16.174** | | **53** | | **4.007** | | **0.000** | | **0.492** | |
| **sOTU richness** | **Threat status (correlation)** | **-0.266** | |  | | **56** | | **-2.007** | | **0.025** | | **-0.266** | |

Whole model: Fisher's C = 1.108; *p* = 0.575; AIC = 15.108
